# Crystal structure of the 4 + 2 cyclo­adduct of photooxidized anthracene and C_60_ fullerene

**DOI:** 10.1107/S1600536814019643

**Published:** 2014-10-31

**Authors:** Gábor Bortel, Éva Kováts, Gábor Oszlányi, Sándor Pekker

**Affiliations:** a Wigner Research Centre for Physics of the Hungarian Academy of Sciences, POB 49, Budapest, H-1525, Hungary; b Rejtő Sándor Faculty of Light Industry and Environmental Engineering, Óbuda University Doberdó út 6, Budapest, H-1034, Hungary

**Keywords:** crystal structure, fullerene, anthracene, photooxidation, cyclo­addition

## Abstract

The structure of the title compound, 5,6-[(1*R*,10*S*)-2,9-dioxatri­cyclo­[8.6.0^3,8^.0^11,16^]hexa­decane-1,10-di­yl]-(C_60_–I_h_)[5,6]fullerene methane­dithione 0.1-solvate, C_74_H_10_O_2_·0.1CS_2_, has tetra­gonal (*P*4_2_/*n*) symmetry at 100 K. It has a unique eight-membered ring, with two incorporated O atoms in place of the original central ring of the anthracene. The distortion of the mol­ecular geometry around the cyclo­adduct bonds corresponds to that seen in related fullerene derivatives. Close packing of the adduct forms cavities partially filled with disordered carbon di­sulfide solvent mol­ecule. The 41% occupancy of the cavities yields an overall 1:0.103 adduct–solvent ratio. Reaction steps are described as light-assisted singlet-oxygen generation, peroxide, epoxide and dioxocin derivative formation and the final step of thermally activated cyclo­addition.

## Abstract 2

The structure of an oxygen-containing cyclo­adduct of C_60_ fullerene and anthracene is presented, features a unique eight-membered ring.

## Chemical context   

The first step of the formation of C_60_(C_14_H_10_O_2_) is the generation of singlet oxygen by the photo-excited C_60_. Singlet oxygen reacts with anthracene *via* several consecutive reactions, similarly to the mechanism described previously by Rigaudy *et al.* (1978 ▶). The addition of singlet oxygen to anthracene results in a highly reactive 9,10-endoperoxide. The thermal rearrangement of the peroxide results in diepoxide, and after a ring extension reaction the final inter­mediare, a [1,4]-dioxocin derivative forms. This inter­mediary reacts with C_60_, forming the product *via* a thermal (4 + 2) cyclo­addition. The cyclo­addition is irreversible because the reverse reaction, the formation of the dioxocin derivative, is energetically unfavourable.
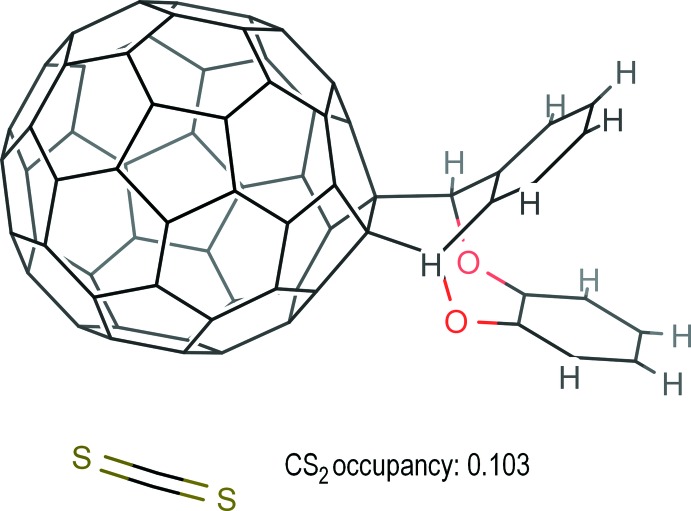


## Structural commentary   

**Figure 1:**
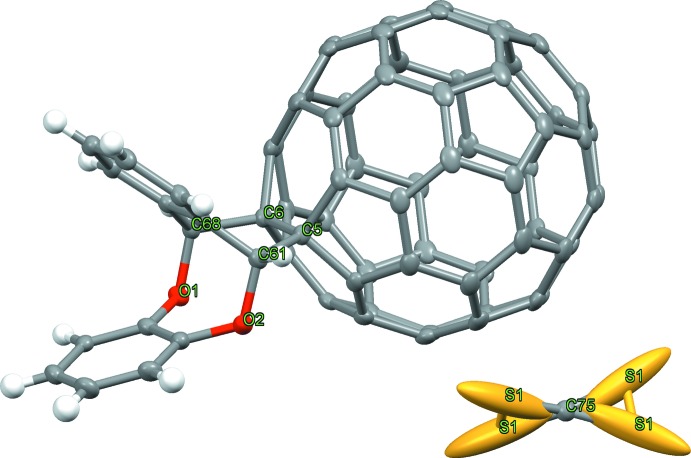
Thermal ellipsoid plot of the cyclo­adduct as a result of unrestrained anisotropic refinement and the disordered solvent.

**Figure 2:**
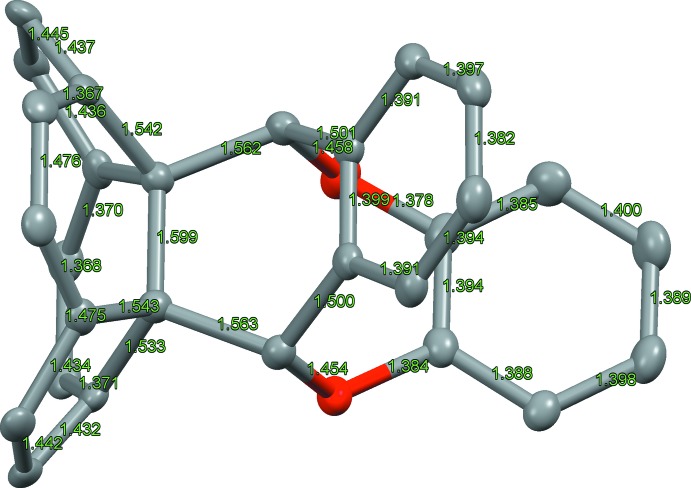
Refined bond lengths around the bonding region of the cyclo­adduct.

The adduct, shows a unique eight-membered ring with two incorporated O atoms. A displacement ellipsoid plot is shown in Fig. 1 ▶. The bond lengths and angles around the strongly distorted bonding region are shown in Figs. 2 ▶ and 3 ▶.

## Supra­molecular features   

**Figure 4:**
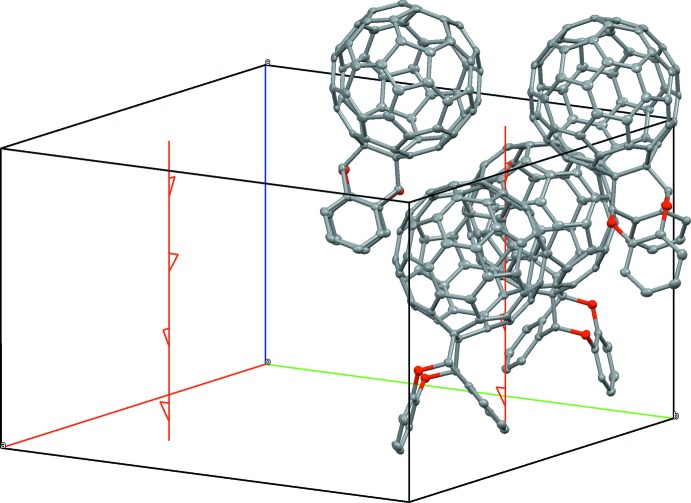
Locally close-packed arrangements of fullerene cages as generated by the 4_2_ screw axis.

**Figure 5:**
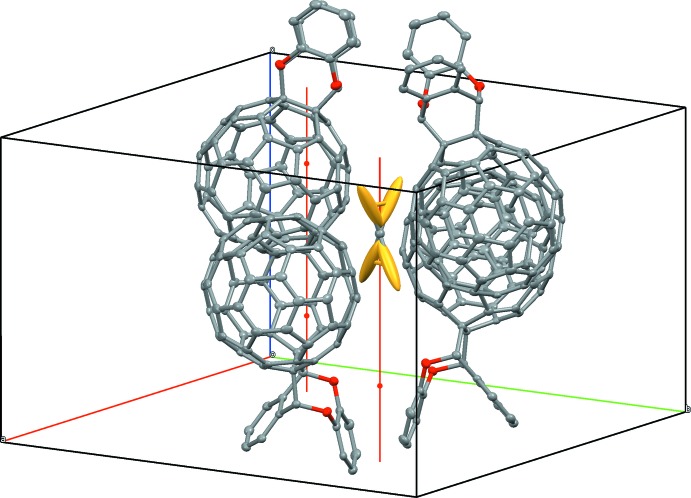
The disordered CS_2_ solvent on a 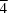 rotoinversion axis surrounded by fullerene cages.

The 10 Å center-to-center distance of the fullerene cages shown in Fig. 4 ▶ indicates a locally realized close packing despite the ligands. The disordered CS_2_ solvent mol­ecules occupy the large cavities between the fullerene adducts, as shown in Fig. 5 ▶.

## Database survey   

No similar eight-membered ring as a substructure of a cyclo­adduct was found in database searches. The structure published here was described in a previous study by Bortel *et al.* (1995 ▶), but neither the collected data nor the refined coordinates are available in that publication. The current structural data collected at low temperature is of significantly better quality in terms of resolution, statistics and number of reflections. The resulting structure is in agreement with the previously determined one.

## Synthesis and crystallization   

C_60_(C_14_H_10_O_2_) was prepared by the photo-oxidation of anthracene and the simultaneous cyclo­addition of its oxidized inter­mediary to C_60_. 144 mg C_60_ (0.2 mmol) and 214 mg anthracene (1.2 mmol) were dissolved in 150 ml toluene. Oxygen was bubbled through the solution and the reaction mixture was illuminated with a luminescent light source of 23 watt. After a reaction time of 1 h at 313 K, the starting materials and the products were separated by column chromatography in silica stationary phase with hexa­ne/toluene eluent mixtures. The major product was recrystallized from carbon di­sulfide by a slow diffusion of iso­pentane into the solution.

## Refinement   

The structure is described in the standard setting of the tetra­gonal space group *P*4_2_/*n* with origin choice 2, origin at inversion center. Least-squares full-matrix refinement on *F* ^2^ with all atoms treated anisotropically (except the C atom of CS_2_) and with riding H atoms without any restraints or constraints (except for a restraint on the bond length of CS_2_) was stable and yielded good figure of merits. The fullerene compound shows no orientational disorder; it is fixed by its attachment. The CS_2_ solvent shows disorder, that was described with an approximate atomic model. The C atom is located on a 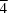 rotoinversion center (2*a* Wyckoff position) and the S-atom positions are ensued by the multiplication effect of the 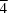 rotoinversion axis. This simple model could not be significantly improved by introducing additional sites or even by masking the corresponding region. The highest peak (1.02 e Å^−3^) and deepest hole (−0.74 e Å^−3^) of the residual electron-density map are located at 0.43 Å from the C and 0.69 Å from the S atom of CS_2_, respectively. Crystal data, data collection and structure refinement details are summarized in Table 1 ▶.

## Supplementary Material

Crystal structure: contains datablock(s) I. DOI: 10.1107/S1600536814019643/zp2014sup1.cif

Structure factors: contains datablock(s) I. DOI: 10.1107/S1600536814019643/zp2014Isup2.hkl

CCDC reference: 1021977

Additional supporting information:  crystallographic information; 3D view; checkCIF report

## Figures and Tables

**Figure 3:**
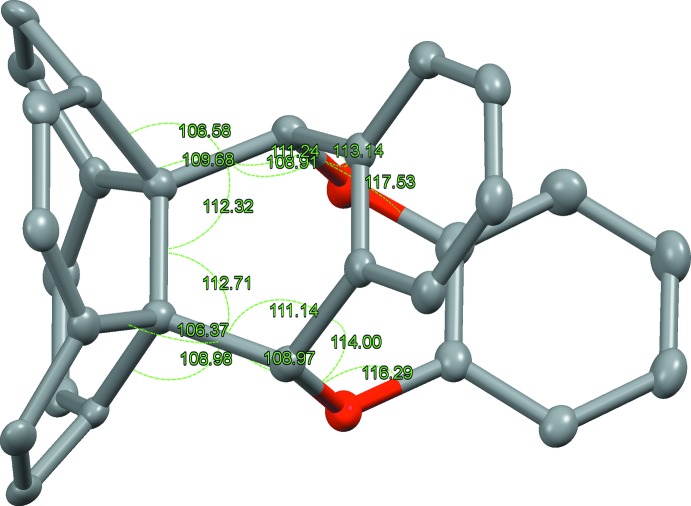
Refined bond angles of the bonding carbon and oxygen atoms.

**Table 1:** Experimental details

Crystal data	
Chemical formula	C_74_H_10_O_2_0.1(CS_2_)
*M*_r_	938.68
Crystal system, space group	Tetragonal, *P*4_2_/*n*
Temperature (K)	100
*a*, *c* ()	22.66570(13), 14.24938(11)
*V* (^3^)	7320.40(10)
*Z*	8
Radiation type	Cu *K*
(mm^1^)	0.91
Crystal size (mm)	0.18 0.15 0.08

Data collection	
Diffractometer	Agilent SuperNova (Dual, Cu at zero, Atlas)
Absorption correction	Multi-scan (*CrysAlis PRO*; Agilent, 2012 ▶)
*T*_min_, *T*_max_	0.911, 1.000
No. of measured, independent and observed [*I* > 2(*I*)] reflections	35247, 7171, 6473
*R*_int_	0.021
(sin /)_max_ (^1^)	0.619

Refinement	
*R*[*F* ^2^ > 2(*F* ^2^)], *wR*(*F* ^2^), *S*	0.045, 0.120, 1.04
No. of reflections	7171
No. of parameters	696
No. of restraints	7
H-atom treatment	H-atom parameters constrained
_max_, _min_ (e ^3^)	1.02, 0.74

Computer programs: *CrysAlis PRO* (Agilent, 2012 ▶), *SHELXS97* and *SHELXL97* (Sheldrick, 2008 ▶), *OLEX2* (Dolomanov *et al.*, 2009 ▶), *Mercury* (Macrae *et al.*, 2008 ▶) and *publCIF* (Westrip, 2010 ▶).

## supplementary crystallographic information

### Crystal data

**Table:**

C_74_H_10_O_2_·0.1(CS_2_)	*D*_x_ = 1.703 Mg m^−^^3^
*M**_r_* = 938.68	Cu *K*α radiation, λ = 1.54184 Å
Tetragonal, *P*4_2_/*n*	Cell parameters from 18450 reflections
*a* = 22.66570 (13) Å	θ = 3.7–72.5°
*c* = 14.24938 (11) Å	µ = 0.91 mm^−^^1^
*V* = 7320.40 (10) Å^3^	*T* = 100 K
*Z* = 8	Irregular, metallic dark black
*F*(000) = 3791	0.18 × 0.15 × 0.08 mm

### Data collection

**Table:**

Agilent SuperNova (Dual, Cu at zero, Atlas) diffractometer	7171 independent reflections
Radiation source: sealed X-ray tube, Agilent SuperNova (Cu) X-ray Source	6473 reflections with *I* > 2σ(*I*)
Mirror monochromator	*R*_int_ = 0.021
Detector resolution: 10.5908 pixels mm^-1^	θ_max_ = 72.6°, θ_min_ = 3.7°
ω scans	*h* = −27→23
Absorption correction: multi-scan (*CrysAlis PRO*; Agilent, 2012)	*k* = −26→27
*T*_min_ = 0.911, *T*_max_ = 1.000	*l* = −17→17
35247 measured reflections	

### Refinement

**Table:**

Refinement on *F*^2^	7 restraints
Least-squares matrix: full	Hydrogen site location: inferred from neighbouring sites
*R*[*F*^2^ > 2σ(*F*^2^)] = 0.045	H-atom parameters constrained
*wR*(*F*^2^) = 0.120	*w* = 1/[σ^2^(*F*_o_^2^) + (0.059*P*)^2^ + 7.0685*P*] where *P* = (*F*_o_^2^ + 2*F*_c_^2^)/3
*S* = 1.04	(Δ/σ)_max_ = 0.003
7171 reflections	Δρ_max_ = 1.02 e Å^−^^3^
696 parameters	Δρ_min_ = −0.74 e Å^−^^3^

### Special details

**Table:**

Experimental. Absorption correction: CrysAlisPro, Agilent Technologies, Empirical absorption correction using spherical harmonics, implemented in SCALE3 ABSPACK scaling algorithm.
Geometry. All e.s.d.'s (except the e.s.d. in the dihedral angle between two l.s. planes) are estimated using the full covariance matrix. The cell e.s.d.'s are taken into account individually in the estimation of e.s.d.'s in distances, angles and torsion angles; correlations between e.s.d.'s in cell parameters are only used when they are defined by crystal symmetry. An approximate (isotropic) treatment of cell e.s.d.'s is used for estimating e.s.d.'s involving l.s. planes.

### Fractional atomic coordinates and isotropic or equivalent isotropic displacement parameters (Å^2^)

**Table:**

	*x*	*y*	*z*	*U*_iso_*/*U*_eq_	Occ. (<1)
O1	0.47684 (5)	0.26998 (5)	0.76855 (8)	0.0210 (3)	
O2	0.37450 (5)	0.21977 (6)	0.70586 (9)	0.0236 (3)	
C1	0.41761 (7)	0.21747 (8)	0.52192 (12)	0.0186 (3)	
C2	0.39498 (7)	0.17973 (8)	0.44940 (12)	0.0210 (4)	
C3	0.41989 (8)	0.12155 (8)	0.46178 (12)	0.0214 (4)	
C4	0.45846 (7)	0.12302 (7)	0.54174 (11)	0.0182 (3)	
C5	0.44903 (7)	0.18069 (7)	0.59712 (12)	0.0176 (3)	
C6	0.50889 (7)	0.20944 (7)	0.63493 (11)	0.0170 (3)	
C7	0.52439 (7)	0.26867 (7)	0.58922 (11)	0.0173 (3)	
C8	0.48633 (8)	0.30127 (7)	0.53539 (12)	0.0199 (3)	
C9	0.43107 (7)	0.27483 (8)	0.50045 (12)	0.0202 (3)	
C10	0.42135 (8)	0.29740 (8)	0.40675 (13)	0.0228 (4)	
C11	0.39726 (8)	0.26139 (8)	0.33720 (13)	0.0237 (4)	
C12	0.38361 (7)	0.20102 (9)	0.35913 (13)	0.0231 (4)	
C13	0.39789 (8)	0.16487 (9)	0.27781 (12)	0.0243 (4)	
C14	0.42198 (8)	0.10914 (8)	0.28984 (13)	0.0241 (4)	
C15	0.43265 (8)	0.08647 (8)	0.38387 (13)	0.0231 (4)	
C16	0.48650 (9)	0.05306 (7)	0.38194 (13)	0.0238 (4)	
C17	0.52473 (8)	0.05630 (7)	0.45803 (12)	0.0224 (4)	
C18	0.50991 (8)	0.09155 (7)	0.53941 (12)	0.0196 (3)	
C19	0.56514 (8)	0.11783 (7)	0.57495 (12)	0.0190 (3)	
C20	0.56485 (7)	0.17411 (7)	0.60967 (11)	0.0172 (3)	
C21	0.61153 (7)	0.21378 (8)	0.58438 (11)	0.0189 (3)	
C22	0.58652 (8)	0.27171 (8)	0.57148 (12)	0.0193 (3)	
C23	0.60939 (8)	0.30951 (7)	0.50312 (12)	0.0214 (4)	
C24	0.56960 (8)	0.34410 (7)	0.44709 (13)	0.0225 (4)	
C25	0.50913 (8)	0.33943 (7)	0.46232 (12)	0.0217 (4)	
C26	0.46911 (8)	0.33716 (7)	0.38330 (13)	0.0235 (4)	
C27	0.49131 (9)	0.33936 (7)	0.29218 (13)	0.0239 (4)	
C28	0.46652 (8)	0.30130 (8)	0.22011 (12)	0.0241 (4)	
C29	0.42054 (8)	0.26311 (8)	0.24215 (12)	0.0236 (4)	
C30	0.42074 (8)	0.20356 (9)	0.20566 (13)	0.0241 (4)	
C31	0.46680 (8)	0.18459 (8)	0.14784 (12)	0.0228 (4)	
C32	0.49167 (8)	0.12613 (8)	0.16037 (12)	0.0233 (4)	
C33	0.46960 (9)	0.08919 (8)	0.23020 (13)	0.0246 (4)	
C34	0.50983 (9)	0.05453 (7)	0.28705 (12)	0.0245 (4)	
C35	0.57045 (9)	0.05839 (7)	0.27278 (13)	0.0241 (4)	
C36	0.61016 (8)	0.06080 (8)	0.35200 (13)	0.0243 (4)	
C37	0.58764 (8)	0.05989 (7)	0.44316 (13)	0.0233 (4)	
C38	0.61245 (8)	0.09803 (8)	0.51441 (12)	0.0219 (4)	
C39	0.65876 (8)	0.13586 (8)	0.49194 (12)	0.0224 (4)	
C40	0.65853 (7)	0.19521 (8)	0.52785 (12)	0.0214 (4)	
C41	0.68151 (7)	0.23398 (8)	0.45576 (12)	0.0225 (4)	
C42	0.65757 (8)	0.28997 (8)	0.44387 (12)	0.0225 (4)	
C43	0.64713 (8)	0.31269 (8)	0.35002 (13)	0.0229 (4)	
C44	0.59253 (9)	0.34602 (7)	0.35189 (13)	0.0236 (4)	
C45	0.55400 (9)	0.34376 (7)	0.27635 (13)	0.0236 (4)	
C46	0.56838 (8)	0.30786 (8)	0.19542 (12)	0.0223 (4)	
C47	0.51416 (8)	0.28177 (8)	0.16034 (12)	0.0224 (4)	
C48	0.51437 (8)	0.22467 (8)	0.12478 (12)	0.0221 (4)	
C49	0.56890 (8)	0.19109 (8)	0.12280 (11)	0.0212 (4)	
C50	0.55485 (8)	0.13015 (8)	0.14525 (12)	0.0221 (4)	
C51	0.59350 (8)	0.09695 (8)	0.20039 (12)	0.0232 (4)	
C52	0.64747 (8)	0.12325 (8)	0.23528 (12)	0.0231 (4)	
C53	0.65811 (8)	0.10066 (8)	0.32865 (13)	0.0243 (4)	
C54	0.68191 (8)	0.13741 (8)	0.39706 (13)	0.0239 (4)	
C55	0.69592 (7)	0.19815 (9)	0.37493 (12)	0.0232 (4)	
C56	0.68558 (7)	0.21988 (8)	0.28473 (12)	0.0230 (4)	
C57	0.66067 (8)	0.27823 (8)	0.27212 (12)	0.0227 (4)	
C58	0.62057 (8)	0.27587 (8)	0.19319 (12)	0.0219 (4)	
C59	0.62078 (8)	0.21610 (8)	0.15649 (12)	0.0207 (4)	
C60	0.66083 (8)	0.18159 (8)	0.21354 (12)	0.0218 (4)	
C61	0.40505 (7)	0.16593 (7)	0.67817 (12)	0.0187 (3)	
H61	0.3755	0.1384	0.6534	0.022*	
C62	0.43589 (7)	0.13605 (7)	0.75835 (12)	0.0182 (3)	
C63	0.41356 (8)	0.08552 (8)	0.80094 (12)	0.0210 (4)	
H63	0.3789	0.0682	0.7793	0.025*	
C64	0.44410 (8)	0.06119 (8)	0.87681 (12)	0.0232 (4)	
H64	0.4299	0.0269	0.9048	0.028*	
C65	0.49495 (8)	0.08705 (8)	0.91104 (12)	0.0237 (4)	
H65	0.5145	0.0705	0.9620	0.028*	
C66	0.51678 (7)	0.13810 (8)	0.86890 (12)	0.0200 (3)	
H66	0.5507	0.1560	0.8921	0.024*	
C67	0.48766 (7)	0.16222 (7)	0.79198 (11)	0.0175 (3)	
C68	0.50944 (7)	0.21694 (7)	0.74399 (12)	0.0178 (3)	
H68	0.5505	0.2230	0.7632	0.021*	
C69	0.43034 (8)	0.26278 (7)	0.83043 (12)	0.0197 (3)	
C70	0.43566 (8)	0.28222 (7)	0.92241 (12)	0.0193 (3)	
H70	0.4702	0.3001	0.9431	0.023*	
C71	0.38791 (8)	0.27441 (8)	0.98321 (13)	0.0252 (4)	
H71	0.3908	0.2871	1.0451	0.030*	
C72	0.33624 (9)	0.24787 (9)	0.95206 (14)	0.0293 (4)	
H72	0.3048	0.2428	0.9933	0.035*	
C73	0.33118 (8)	0.22874 (9)	0.85896 (14)	0.0264 (4)	
H73	0.2966	0.2110	0.8380	0.032*	
C74	0.37838 (8)	0.23669 (8)	0.79852 (12)	0.0215 (4)	
S1	0.2763 (3)	0.2431 (3)	0.1529 (6)	0.264 (6)	0.206 (3)
C75	0.2500	0.2500	0.2500	0.022 (2)*	0.413 (6)

### Atomic displacement parameters (Å^2^)

**Table:**

	*U*^11^	*U*^22^	*U*^33^	*U*^12^	*U*^13^	*U*^23^
O1	0.0251 (6)	0.0179 (6)	0.0199 (6)	0.0006 (5)	0.0040 (5)	−0.0009 (5)
O2	0.0226 (6)	0.0271 (7)	0.0210 (6)	0.0059 (5)	−0.0028 (5)	−0.0026 (5)
C1	0.0129 (7)	0.0264 (9)	0.0165 (8)	0.0013 (6)	0.0021 (6)	0.0008 (7)
C2	0.0124 (7)	0.0308 (9)	0.0198 (8)	−0.0047 (7)	0.0007 (6)	0.0008 (7)
C3	0.0184 (8)	0.0259 (9)	0.0201 (8)	−0.0118 (7)	0.0009 (7)	0.0016 (7)
C4	0.0212 (8)	0.0176 (8)	0.0157 (8)	−0.0067 (6)	0.0024 (6)	0.0021 (6)
C5	0.0174 (8)	0.0191 (8)	0.0165 (8)	−0.0019 (6)	0.0001 (6)	0.0003 (6)
C6	0.0176 (8)	0.0173 (8)	0.0162 (8)	−0.0011 (6)	−0.0010 (6)	0.0000 (6)
C7	0.0221 (8)	0.0166 (8)	0.0131 (7)	−0.0031 (6)	0.0014 (6)	−0.0044 (6)
C8	0.0262 (9)	0.0143 (8)	0.0190 (8)	0.0027 (6)	0.0041 (7)	−0.0035 (6)
C9	0.0187 (8)	0.0219 (8)	0.0199 (8)	0.0073 (7)	0.0022 (6)	−0.0010 (7)
C10	0.0211 (8)	0.0236 (9)	0.0238 (9)	0.0100 (7)	0.0011 (7)	0.0030 (7)
C11	0.0162 (8)	0.0319 (10)	0.0230 (9)	0.0075 (7)	−0.0031 (7)	0.0040 (7)
C12	0.0121 (8)	0.0351 (10)	0.0221 (9)	−0.0020 (7)	−0.0030 (6)	0.0011 (7)
C13	0.0162 (8)	0.0376 (10)	0.0192 (8)	−0.0081 (7)	−0.0061 (7)	−0.0002 (7)
C14	0.0231 (9)	0.0276 (9)	0.0215 (9)	−0.0141 (7)	−0.0028 (7)	−0.0040 (7)
C15	0.0245 (9)	0.0217 (8)	0.0231 (9)	−0.0148 (7)	0.0003 (7)	−0.0022 (7)
C16	0.0367 (10)	0.0122 (8)	0.0226 (9)	−0.0089 (7)	0.0031 (7)	−0.0010 (6)
C17	0.0356 (10)	0.0105 (7)	0.0211 (8)	−0.0015 (7)	0.0038 (7)	0.0038 (6)
C18	0.0274 (9)	0.0138 (7)	0.0175 (8)	−0.0029 (6)	0.0010 (7)	0.0047 (6)
C19	0.0222 (8)	0.0193 (8)	0.0154 (8)	0.0036 (6)	−0.0012 (6)	0.0063 (6)
C20	0.0166 (8)	0.0227 (8)	0.0123 (7)	0.0003 (6)	−0.0026 (6)	0.0018 (6)
C21	0.0175 (8)	0.0258 (9)	0.0133 (7)	−0.0028 (6)	−0.0053 (6)	−0.0004 (6)
C22	0.0213 (8)	0.0218 (8)	0.0147 (8)	−0.0061 (6)	−0.0019 (6)	−0.0050 (6)
C23	0.0255 (9)	0.0193 (8)	0.0194 (8)	−0.0104 (7)	−0.0009 (7)	−0.0043 (7)
C24	0.0330 (10)	0.0117 (7)	0.0227 (9)	−0.0065 (7)	0.0021 (7)	−0.0032 (6)
C25	0.0334 (10)	0.0111 (7)	0.0207 (8)	0.0034 (7)	0.0037 (7)	−0.0020 (6)
C26	0.0297 (9)	0.0141 (8)	0.0265 (9)	0.0084 (7)	0.0019 (7)	0.0030 (7)
C27	0.0335 (10)	0.0147 (8)	0.0236 (9)	0.0066 (7)	−0.0011 (7)	0.0065 (7)
C28	0.0293 (9)	0.0236 (9)	0.0195 (8)	0.0079 (7)	−0.0028 (7)	0.0078 (7)
C29	0.0206 (8)	0.0313 (10)	0.0190 (8)	0.0061 (7)	−0.0064 (7)	0.0049 (7)
C30	0.0184 (8)	0.0351 (10)	0.0188 (8)	−0.0023 (7)	−0.0078 (7)	0.0020 (7)
C31	0.0250 (9)	0.0309 (9)	0.0125 (7)	−0.0046 (7)	−0.0059 (7)	0.0004 (7)
C32	0.0292 (9)	0.0269 (9)	0.0137 (8)	−0.0069 (7)	−0.0018 (7)	−0.0055 (7)
C33	0.0323 (10)	0.0228 (9)	0.0188 (8)	−0.0120 (7)	−0.0027 (7)	−0.0063 (7)
C34	0.0396 (11)	0.0132 (8)	0.0207 (9)	−0.0069 (7)	0.0022 (8)	−0.0051 (6)
C35	0.0392 (10)	0.0120 (8)	0.0212 (8)	0.0030 (7)	0.0048 (8)	−0.0043 (6)
C36	0.0322 (10)	0.0144 (8)	0.0264 (9)	0.0094 (7)	0.0051 (8)	−0.0006 (7)
C37	0.0327 (10)	0.0133 (8)	0.0240 (9)	0.0076 (7)	0.0029 (7)	0.0039 (7)
C38	0.0255 (9)	0.0215 (8)	0.0186 (8)	0.0096 (7)	−0.0013 (7)	0.0056 (7)
C39	0.0176 (8)	0.0308 (9)	0.0190 (8)	0.0082 (7)	−0.0022 (7)	0.0041 (7)
C40	0.0139 (8)	0.0332 (10)	0.0172 (8)	−0.0005 (7)	−0.0050 (6)	0.0020 (7)
C41	0.0126 (8)	0.0351 (10)	0.0197 (8)	−0.0061 (7)	−0.0020 (6)	−0.0008 (7)
C42	0.0190 (8)	0.0277 (9)	0.0207 (8)	−0.0127 (7)	−0.0015 (7)	−0.0032 (7)
C43	0.0240 (9)	0.0215 (8)	0.0233 (9)	−0.0134 (7)	0.0030 (7)	−0.0005 (7)
C44	0.0334 (10)	0.0137 (8)	0.0239 (9)	−0.0090 (7)	0.0040 (7)	0.0008 (7)
C45	0.0365 (10)	0.0126 (8)	0.0217 (9)	−0.0026 (7)	0.0040 (7)	0.0054 (6)
C46	0.0320 (10)	0.0181 (8)	0.0166 (8)	−0.0028 (7)	0.0020 (7)	0.0069 (6)
C47	0.0274 (9)	0.0251 (9)	0.0148 (8)	0.0021 (7)	−0.0018 (7)	0.0088 (7)
C48	0.0259 (9)	0.0284 (9)	0.0120 (8)	−0.0016 (7)	−0.0037 (6)	0.0034 (7)
C49	0.0272 (9)	0.0246 (9)	0.0119 (7)	−0.0019 (7)	0.0025 (6)	−0.0012 (6)
C50	0.0305 (9)	0.0222 (9)	0.0135 (8)	−0.0034 (7)	0.0027 (7)	−0.0051 (6)
C51	0.0323 (10)	0.0192 (8)	0.0181 (8)	0.0016 (7)	0.0068 (7)	−0.0059 (7)
C52	0.0237 (9)	0.0261 (9)	0.0194 (8)	0.0086 (7)	0.0066 (7)	−0.0019 (7)
C53	0.0239 (9)	0.0245 (9)	0.0243 (9)	0.0105 (7)	0.0054 (7)	0.0010 (7)
C54	0.0156 (8)	0.0316 (10)	0.0243 (9)	0.0084 (7)	0.0015 (7)	0.0021 (7)
C55	0.0120 (8)	0.0354 (10)	0.0223 (9)	0.0001 (7)	0.0014 (6)	0.0000 (7)
C56	0.0149 (8)	0.0318 (10)	0.0223 (9)	−0.0042 (7)	0.0051 (7)	−0.0003 (7)
C57	0.0206 (8)	0.0279 (9)	0.0195 (8)	−0.0112 (7)	0.0037 (7)	0.0031 (7)
C58	0.0258 (9)	0.0232 (9)	0.0166 (8)	−0.0066 (7)	0.0048 (7)	0.0048 (7)
C59	0.0240 (9)	0.0244 (9)	0.0137 (8)	−0.0015 (7)	0.0064 (6)	0.0018 (6)
C60	0.0185 (8)	0.0304 (9)	0.0166 (8)	0.0009 (7)	0.0056 (6)	−0.0014 (7)
C61	0.0163 (8)	0.0209 (8)	0.0188 (8)	−0.0024 (6)	0.0009 (6)	−0.0014 (6)
C62	0.0184 (8)	0.0197 (8)	0.0165 (8)	0.0001 (6)	0.0039 (6)	−0.0023 (6)
C63	0.0249 (9)	0.0193 (8)	0.0189 (8)	−0.0029 (7)	0.0033 (7)	−0.0023 (7)
C64	0.0281 (9)	0.0202 (8)	0.0214 (9)	−0.0007 (7)	0.0070 (7)	0.0018 (7)
C65	0.0262 (9)	0.0273 (9)	0.0178 (8)	0.0062 (7)	0.0015 (7)	0.0020 (7)
C66	0.0175 (8)	0.0254 (9)	0.0171 (8)	0.0030 (6)	0.0017 (6)	−0.0022 (7)
C67	0.0177 (8)	0.0191 (8)	0.0156 (8)	0.0017 (6)	0.0032 (6)	−0.0020 (6)
C68	0.0172 (8)	0.0199 (8)	0.0164 (8)	−0.0004 (6)	−0.0008 (6)	−0.0016 (6)
C69	0.0225 (8)	0.0164 (8)	0.0201 (8)	0.0038 (6)	0.0031 (7)	0.0008 (6)
C70	0.0227 (8)	0.0167 (8)	0.0184 (8)	0.0054 (6)	−0.0020 (7)	0.0002 (6)
C71	0.0316 (10)	0.0248 (9)	0.0192 (8)	0.0078 (7)	0.0021 (7)	−0.0027 (7)
C72	0.0255 (9)	0.0347 (10)	0.0278 (10)	0.0073 (8)	0.0082 (8)	−0.0030 (8)
C73	0.0182 (8)	0.0302 (10)	0.0309 (10)	0.0045 (7)	0.0003 (7)	−0.0034 (8)
C74	0.0231 (9)	0.0220 (8)	0.0193 (8)	0.0068 (7)	−0.0011 (7)	−0.0010 (7)
S1	0.210 (8)	0.061 (4)	0.522 (14)	−0.049 (5)	−0.292 (9)	0.082 (7)

### Geometric parameters (Å, º)

**Table:**

O1—C68	1.454 (2)	C32—C33	1.393 (3)
O1—C69	1.384 (2)	C32—C50	1.451 (3)
O2—C61	1.458 (2)	C33—C34	1.451 (3)
O2—C74	1.378 (2)	C34—C35	1.392 (3)
C1—C2	1.436 (2)	C35—C36	1.445 (3)
C1—C5	1.533 (2)	C35—C51	1.449 (2)
C1—C9	1.370 (2)	C36—C37	1.396 (3)
C2—C3	1.445 (3)	C36—C53	1.452 (3)
C2—C12	1.398 (2)	C37—C38	1.447 (3)
C3—C4	1.437 (2)	C38—C39	1.393 (3)
C3—C15	1.396 (3)	C39—C40	1.439 (3)
C4—C5	1.542 (2)	C39—C54	1.451 (2)
C4—C18	1.368 (2)	C40—C41	1.449 (2)
C5—C6	1.599 (2)	C41—C42	1.391 (3)
C5—C61	1.562 (2)	C41—C55	1.447 (3)
C6—C7	1.533 (2)	C42—C43	1.452 (3)
C6—C20	1.543 (2)	C43—C44	1.450 (3)
C6—C68	1.563 (2)	C43—C57	1.391 (3)
C7—C8	1.371 (2)	C44—C45	1.387 (3)
C7—C22	1.432 (2)	C45—C46	1.449 (3)
C8—C9	1.475 (2)	C46—C47	1.453 (3)
C8—C25	1.449 (2)	C46—C58	1.388 (3)
C9—C10	1.447 (2)	C47—C48	1.390 (3)
C10—C11	1.395 (3)	C48—C49	1.452 (3)
C10—C26	1.448 (3)	C49—C50	1.453 (2)
C11—C12	1.437 (3)	C49—C59	1.391 (3)
C11—C29	1.454 (3)	C50—C51	1.397 (3)
C12—C13	1.456 (3)	C51—C52	1.449 (3)
C13—C14	1.387 (3)	C52—C53	1.446 (3)
C13—C30	1.447 (3)	C52—C60	1.391 (3)
C14—C15	1.455 (3)	C53—C54	1.391 (3)
C14—C33	1.446 (3)	C54—C55	1.448 (3)
C15—C16	1.437 (3)	C55—C56	1.396 (3)
C16—C17	1.390 (3)	C56—C57	1.449 (3)
C16—C34	1.452 (2)	C56—C60	1.448 (3)
C17—C18	1.448 (2)	C57—C58	1.447 (3)
C17—C37	1.444 (3)	C58—C59	1.452 (2)
C18—C19	1.476 (2)	C59—C60	1.448 (3)
C19—C20	1.368 (2)	C61—C62	1.501 (2)
C19—C38	1.448 (2)	C62—C63	1.392 (2)
C20—C21	1.434 (2)	C62—C67	1.400 (2)
C21—C22	1.442 (2)	C63—C64	1.397 (3)
C21—C40	1.400 (2)	C64—C65	1.382 (3)
C22—C23	1.397 (2)	C65—C66	1.394 (3)
C23—C24	1.437 (3)	C66—C67	1.391 (2)
C23—C42	1.450 (3)	C67—C68	1.500 (2)
C24—C25	1.392 (3)	C69—C70	1.388 (2)
C24—C44	1.453 (2)	C69—C74	1.394 (3)
C25—C26	1.447 (3)	C70—C71	1.397 (3)
C26—C27	1.393 (3)	C71—C72	1.389 (3)
C27—C28	1.454 (3)	C72—C73	1.400 (3)
C27—C45	1.442 (3)	C73—C74	1.385 (3)
C28—C29	1.391 (3)	S1—S1^i^	1.234 (13)
C28—C47	1.445 (3)	S1—C75	1.514 (9)
C29—C30	1.446 (3)	C75—S1^ii^	1.514 (9)
C30—C31	1.398 (3)	C75—S1^iii^	1.514 (9)
C31—C32	1.451 (3)	C75—S1^i^	1.514 (9)
C31—C48	1.448 (3)		
			
C69—O1—C68	116.28 (12)	C34—C35—C51	119.86 (17)
C74—O2—C61	117.52 (13)	C36—C35—C51	107.97 (16)
C2—C1—C5	110.18 (15)	C35—C36—C53	108.11 (16)
C9—C1—C2	118.95 (16)	C37—C36—C35	119.92 (17)
C9—C1—C5	124.67 (15)	C37—C36—C53	119.74 (17)
C1—C2—C3	108.43 (15)	C17—C37—C38	108.33 (15)
C12—C2—C1	121.49 (16)	C36—C37—C17	119.90 (17)
C12—C2—C3	119.95 (16)	C36—C37—C38	120.14 (17)
C4—C3—C2	108.26 (15)	C37—C38—C19	108.39 (16)
C15—C3—C2	120.25 (16)	C39—C38—C19	120.29 (16)
C15—C3—C4	121.20 (17)	C39—C38—C37	119.96 (16)
C3—C4—C5	109.96 (15)	C38—C39—C40	119.40 (16)
C18—C4—C3	119.18 (16)	C38—C39—C54	120.12 (17)
C18—C4—C5	124.95 (15)	C40—C39—C54	108.07 (16)
C1—C5—C4	99.65 (13)	C21—C40—C39	119.23 (16)
C1—C5—C6	114.08 (13)	C21—C40—C41	119.94 (17)
C1—C5—C61	109.69 (13)	C39—C40—C41	108.27 (15)
C4—C5—C6	113.61 (13)	C42—C41—C40	119.98 (16)
C4—C5—C61	106.58 (13)	C42—C41—C55	120.22 (16)
C61—C5—C6	112.32 (13)	C55—C41—C40	107.77 (16)
C7—C6—C5	114.10 (13)	C23—C42—C43	107.77 (16)
C7—C6—C20	99.62 (13)	C41—C42—C23	120.12 (16)
C7—C6—C68	108.99 (13)	C41—C42—C43	119.96 (16)
C20—C6—C5	114.05 (13)	C44—C43—C42	107.86 (15)
C20—C6—C68	106.38 (13)	C57—C43—C42	119.96 (17)
C68—C6—C5	112.71 (13)	C57—C43—C44	119.69 (17)
C8—C7—C6	124.46 (15)	C43—C44—C24	107.87 (16)
C8—C7—C22	119.60 (15)	C45—C44—C24	119.87 (17)
C22—C7—C6	110.02 (14)	C45—C44—C43	120.25 (16)
C7—C8—C9	120.28 (15)	C27—C45—C46	107.90 (16)
C7—C8—C25	119.98 (16)	C44—C45—C27	120.10 (16)
C25—C8—C9	107.62 (15)	C44—C45—C46	119.79 (17)
C1—C9—C8	119.94 (15)	C45—C46—C47	108.20 (16)
C1—C9—C10	120.51 (16)	C58—C46—C45	120.24 (17)
C10—C9—C8	107.29 (15)	C58—C46—C47	120.04 (16)
C9—C10—C26	108.62 (16)	C28—C47—C46	107.74 (16)
C11—C10—C9	120.56 (17)	C48—C47—C28	120.18 (17)
C11—C10—C26	119.53 (17)	C48—C47—C46	120.11 (16)
C10—C11—C12	119.13 (16)	C31—C48—C49	108.02 (16)
C10—C11—C29	120.26 (17)	C47—C48—C31	119.94 (17)
C12—C11—C29	107.82 (16)	C47—C48—C49	119.89 (16)
C2—C12—C11	119.27 (16)	C48—C49—C50	107.91 (15)
C2—C12—C13	119.81 (17)	C59—C49—C48	119.95 (16)
C11—C12—C13	108.36 (16)	C59—C49—C50	119.79 (16)
C14—C13—C12	120.13 (16)	C32—C50—C49	107.99 (16)
C14—C13—C30	119.93 (17)	C51—C50—C32	120.10 (16)
C30—C13—C12	107.70 (16)	C51—C50—C49	119.89 (16)
C13—C14—C15	120.06 (17)	C50—C51—C35	119.93 (17)
C13—C14—C33	120.40 (16)	C50—C51—C52	120.06 (16)
C33—C14—C15	107.85 (17)	C52—C51—C35	107.96 (16)
C3—C15—C14	119.77 (17)	C53—C52—C51	108.11 (16)
C3—C15—C16	119.44 (16)	C60—C52—C51	119.90 (16)
C16—C15—C14	108.04 (16)	C60—C52—C53	120.35 (17)
C15—C16—C34	108.37 (16)	C52—C53—C36	107.84 (16)
C17—C16—C15	119.14 (16)	C54—C53—C36	120.15 (16)
C17—C16—C34	119.87 (17)	C54—C53—C52	119.83 (17)
C16—C17—C18	120.62 (17)	C53—C54—C39	119.89 (17)
C16—C17—C37	120.28 (16)	C53—C54—C55	120.12 (17)
C37—C17—C18	108.40 (16)	C55—C54—C39	107.78 (16)
C4—C18—C17	120.35 (16)	C41—C55—C54	108.12 (15)
C4—C18—C19	120.29 (15)	C56—C55—C41	119.80 (17)
C17—C18—C19	107.50 (15)	C56—C55—C54	119.95 (17)
C20—C19—C18	119.76 (15)	C55—C56—C57	120.10 (16)
C20—C19—C38	120.54 (16)	C55—C56—C60	119.89 (17)
C38—C19—C18	107.37 (15)	C60—C56—C57	108.01 (15)
C19—C20—C6	124.91 (15)	C43—C57—C56	119.95 (16)
C19—C20—C21	119.34 (15)	C43—C57—C58	120.14 (17)
C21—C20—C6	109.86 (14)	C58—C57—C56	107.90 (15)
C20—C21—C22	108.23 (14)	C46—C58—C57	119.88 (16)
C40—C21—C20	121.14 (16)	C46—C58—C59	119.89 (16)
C40—C21—C22	119.97 (16)	C57—C58—C59	108.20 (16)
C7—C22—C21	108.68 (14)	C49—C59—C58	120.12 (16)
C23—C22—C7	121.16 (16)	C49—C59—C60	120.24 (16)
C23—C22—C21	120.09 (16)	C60—C59—C58	107.68 (15)
C22—C23—C24	119.27 (16)	C52—C60—C56	119.86 (16)
C22—C23—C42	119.89 (16)	C52—C60—C59	120.12 (16)
C24—C23—C42	108.42 (15)	C59—C60—C56	108.21 (16)
C23—C24—C44	108.08 (16)	O2—C61—C5	108.91 (13)
C25—C24—C23	119.34 (16)	O2—C61—C62	113.15 (13)
C25—C24—C44	120.00 (17)	C62—C61—C5	111.24 (13)
C24—C25—C8	120.57 (16)	C63—C62—C61	122.28 (15)
C24—C25—C26	119.93 (16)	C63—C62—C67	120.30 (16)
C26—C25—C8	108.33 (16)	C67—C62—C61	117.38 (15)
C25—C26—C10	108.14 (15)	C62—C63—C64	118.82 (16)
C27—C26—C10	120.50 (17)	C65—C64—C63	121.26 (16)
C27—C26—C25	119.82 (17)	C64—C65—C66	119.73 (16)
C26—C27—C28	119.81 (17)	C67—C66—C65	119.81 (16)
C26—C27—C45	120.27 (17)	C62—C67—C68	118.05 (15)
C45—C27—C28	108.14 (16)	C66—C67—C62	120.06 (16)
C29—C28—C27	119.96 (16)	C66—C67—C68	121.88 (15)
C29—C28—C47	120.17 (17)	O1—C68—C6	108.98 (13)
C47—C28—C27	108.01 (16)	O1—C68—C67	114.01 (13)
C28—C29—C11	119.93 (17)	C67—C68—C6	111.14 (13)
C28—C29—C30	119.82 (17)	O1—C69—C70	119.87 (16)
C30—C29—C11	108.12 (16)	O1—C69—C74	119.06 (15)
C29—C30—C13	107.99 (16)	C70—C69—C74	121.06 (16)
C31—C30—C13	119.98 (17)	C69—C70—C71	118.55 (16)
C31—C30—C29	120.09 (17)	C72—C71—C70	120.64 (17)
C30—C31—C32	119.90 (16)	C71—C72—C73	120.37 (17)
C30—C31—C48	119.80 (17)	C74—C73—C72	119.04 (18)
C48—C31—C32	108.16 (16)	O2—C74—C69	118.98 (16)
C31—C32—C50	107.92 (15)	O2—C74—C73	120.69 (16)
C33—C32—C31	119.82 (17)	C73—C74—C69	120.32 (17)
C33—C32—C50	119.87 (17)	S1^i^—S1—C75	65.96 (19)
C14—C33—C34	108.08 (16)	S1—C75—S1^ii^	146.5 (3)
C32—C33—C14	119.97 (17)	S1^ii^—C75—S1^iii^	48.1 (4)
C32—C33—C34	119.91 (17)	S1—C75—S1^iii^	146.5 (3)
C33—C34—C16	107.67 (17)	S1—C75—S1^i^	48.1 (4)
C35—C34—C16	119.80 (17)	S1^i^—C75—S1^iii^	146.5 (3)
C35—C34—C33	120.32 (16)	S1^ii^—C75—S1^i^	146.5 (3)
C34—C35—C36	120.22 (16)		
			
O1—C69—C70—C71	179.91 (15)	C28—C29—C30—C13	142.22 (16)
O1—C69—C74—O2	−1.1 (2)	C28—C29—C30—C31	−0.5 (3)
O1—C69—C74—C73	179.79 (16)	C28—C47—C48—C31	−0.1 (2)
O2—C61—C62—C63	104.02 (18)	C28—C47—C48—C49	−138.10 (17)
O2—C61—C62—C67	−73.64 (18)	C29—C11—C12—C2	141.49 (16)
C1—C2—C3—C4	−0.39 (19)	C29—C11—C12—C13	−0.37 (19)
C1—C2—C3—C15	−145.85 (16)	C29—C28—C47—C46	−142.88 (16)
C1—C2—C12—C11	2.7 (3)	C29—C28—C47—C48	−0.2 (3)
C1—C2—C12—C13	140.25 (17)	C29—C30—C31—C32	138.21 (17)
C1—C5—C6—C7	−0.10 (19)	C29—C30—C31—C48	0.2 (3)
C1—C5—C6—C20	−113.68 (15)	C30—C13—C14—C15	138.15 (17)
C1—C5—C6—C68	124.89 (15)	C30—C13—C14—C33	−0.5 (2)
C1—C5—C61—O2	−48.00 (17)	C30—C31—C32—C33	−0.2 (3)
C1—C5—C61—C62	−173.37 (14)	C30—C31—C32—C50	−142.23 (16)
C1—C9—C10—C11	1.0 (3)	C30—C31—C48—C47	0.1 (2)
C1—C9—C10—C26	−142.38 (16)	C30—C31—C48—C49	142.52 (16)
C2—C1—C5—C4	17.70 (16)	C31—C32—C33—C14	0.1 (3)
C2—C1—C5—C6	139.10 (14)	C31—C32—C33—C34	−138.06 (17)
C2—C1—C5—C61	−93.91 (16)	C31—C32—C50—C49	−0.34 (19)
C2—C1—C9—C8	−136.12 (17)	C31—C32—C50—C51	142.21 (16)
C2—C1—C9—C10	1.4 (2)	C31—C48—C49—C50	−0.31 (18)
C2—C3—C4—C5	12.28 (18)	C31—C48—C49—C59	−142.38 (16)
C2—C3—C4—C18	−142.00 (16)	C32—C31—C48—C47	−142.27 (16)
C2—C3—C15—C14	1.3 (2)	C32—C31—C48—C49	0.10 (19)
C2—C3—C15—C16	138.43 (17)	C32—C33—C34—C16	142.82 (16)
C2—C12—C13—C14	0.9 (3)	C32—C33—C34—C35	0.6 (3)
C2—C12—C13—C30	−141.38 (16)	C32—C50—C51—C35	0.0 (2)
C3—C2—C12—C11	−138.60 (17)	C32—C50—C51—C52	−138.06 (17)
C3—C2—C12—C13	−1.1 (2)	C33—C14—C15—C3	141.64 (16)
C3—C4—C5—C1	−17.93 (16)	C33—C14—C15—C16	0.20 (19)
C3—C4—C5—C6	−139.67 (14)	C33—C32—C50—C49	−142.39 (16)
C3—C4—C5—C61	96.10 (15)	C33—C32—C50—C51	0.2 (3)
C3—C4—C18—C17	−1.2 (2)	C33—C34—C35—C36	137.88 (17)
C3—C4—C18—C19	137.04 (16)	C33—C34—C35—C51	−0.4 (2)
C3—C15—C16—C17	0.2 (2)	C34—C16—C17—C18	139.09 (17)
C3—C15—C16—C34	−141.63 (16)	C34—C16—C17—C37	−1.3 (2)
C4—C3—C15—C14	−139.66 (17)	C34—C35—C36—C37	−0.7 (2)
C4—C3—C15—C16	−2.6 (2)	C34—C35—C36—C53	−142.92 (16)
C4—C5—C6—C7	113.21 (15)	C34—C35—C51—C50	0.1 (2)
C4—C5—C6—C20	−0.36 (19)	C34—C35—C51—C52	142.65 (16)
C4—C5—C6—C68	−121.80 (15)	C35—C36—C37—C17	0.3 (2)
C4—C5—C61—O2	−155.00 (13)	C35—C36—C37—C38	−138.62 (17)
C4—C5—C61—C62	79.63 (16)	C35—C36—C53—C52	0.62 (19)
C4—C18—C19—C20	−0.5 (2)	C35—C36—C53—C54	142.91 (17)
C4—C18—C19—C38	−142.91 (16)	C35—C51—C52—C53	0.52 (19)
C5—C1—C2—C3	−11.72 (18)	C35—C51—C52—C60	−142.72 (16)
C5—C1—C2—C12	−156.93 (15)	C36—C35—C51—C50	−142.72 (16)
C5—C1—C9—C8	13.4 (3)	C36—C35—C51—C52	−0.13 (19)
C5—C1—C9—C10	150.92 (16)	C36—C37—C38—C19	143.83 (16)
C5—C4—C18—C17	−151.33 (16)	C36—C37—C38—C39	0.2 (2)
C5—C4—C18—C19	−13.1 (2)	C36—C53—C54—C39	0.0 (3)
C5—C6—C7—C8	12.8 (2)	C36—C53—C54—C55	−137.75 (17)
C5—C6—C7—C22	−139.76 (14)	C37—C17—C18—C4	143.34 (16)
C5—C6—C20—C19	−12.5 (2)	C37—C17—C18—C19	0.45 (18)
C5—C6—C20—C21	140.04 (14)	C37—C36—C53—C52	−141.69 (17)
C5—C6—C68—O1	−80.86 (16)	C37—C36—C53—C54	0.6 (3)
C5—C6—C68—C67	45.61 (18)	C37—C38—C39—C40	138.02 (17)
C5—C61—C62—C63	−133.00 (16)	C37—C38—C39—C54	0.4 (2)
C5—C61—C62—C67	49.3 (2)	C38—C19—C20—C6	151.10 (16)
C6—C5—C61—O2	79.98 (16)	C38—C19—C20—C21	0.9 (2)
C6—C5—C61—C62	−45.40 (18)	C38—C39—C40—C21	−0.3 (2)
C6—C7—C8—C9	−13.6 (2)	C38—C39—C40—C41	−142.19 (16)
C6—C7—C8—C25	−151.41 (16)	C38—C39—C54—C53	−0.6 (3)
C6—C7—C22—C21	11.84 (18)	C38—C39—C54—C55	141.84 (16)
C6—C7—C22—C23	157.20 (15)	C39—C40—C41—C42	142.71 (16)
C6—C20—C21—C22	−12.40 (18)	C39—C40—C41—C55	0.05 (19)
C6—C20—C21—C40	−156.85 (15)	C39—C54—C55—C41	0.02 (19)
C7—C6—C20—C19	−134.45 (16)	C39—C54—C55—C56	−142.36 (16)
C7—C6—C20—C21	18.10 (16)	C40—C21—C22—C7	145.34 (15)
C7—C6—C68—O1	46.87 (17)	C40—C21—C22—C23	−0.5 (2)
C7—C6—C68—C67	173.34 (13)	C40—C39—C54—C53	−142.38 (17)
C7—C8—C9—C1	0.1 (2)	C40—C39—C54—C55	0.01 (19)
C7—C8—C9—C10	−142.38 (16)	C40—C41—C42—C23	−0.4 (2)
C7—C8—C25—C24	−1.0 (2)	C40—C41—C42—C43	−138.24 (17)
C7—C8—C25—C26	142.66 (16)	C40—C41—C55—C54	−0.04 (19)
C7—C22—C23—C24	−2.6 (2)	C40—C41—C55—C56	142.40 (16)
C7—C22—C23—C42	−140.30 (17)	C41—C42—C43—C44	142.36 (16)
C8—C7—C22—C21	−142.28 (15)	C41—C42—C43—C57	0.5 (2)
C8—C7—C22—C23	3.1 (2)	C41—C55—C56—C57	0.2 (2)
C8—C9—C10—C11	143.24 (16)	C41—C55—C56—C60	−138.00 (17)
C8—C9—C10—C26	−0.17 (18)	C42—C23—C24—C25	142.32 (16)
C8—C25—C26—C10	−0.21 (19)	C42—C23—C24—C44	0.43 (18)
C8—C25—C26—C27	−143.67 (16)	C42—C41—C55—C54	−142.60 (16)
C9—C1—C2—C3	141.89 (16)	C42—C41—C55—C56	−0.2 (2)
C9—C1—C2—C12	−3.3 (2)	C42—C43—C44—C24	0.37 (18)
C9—C1—C5—C4	−134.07 (17)	C42—C43—C44—C45	−142.17 (16)
C9—C1—C5—C6	−12.7 (2)	C42—C43—C57—C56	−0.5 (2)
C9—C1—C5—C61	114.31 (18)	C42—C43—C57—C58	137.69 (17)
C9—C8—C25—C24	−143.61 (16)	C43—C44—C45—C27	137.96 (17)
C9—C8—C25—C26	0.10 (18)	C43—C44—C45—C46	0.1 (2)
C9—C10—C11—C12	−1.6 (3)	C43—C57—C58—C46	−0.3 (2)
C9—C10—C11—C29	−138.57 (17)	C43—C57—C58—C59	−142.84 (16)
C9—C10—C26—C25	0.24 (19)	C44—C24—C25—C8	138.90 (17)
C9—C10—C26—C27	143.41 (16)	C44—C24—C25—C26	−0.7 (2)
C10—C11—C12—C2	−0.2 (3)	C44—C43—C57—C56	−137.88 (17)
C10—C11—C12—C13	−142.12 (16)	C44—C43—C57—C58	0.3 (2)
C10—C11—C29—C28	−0.8 (3)	C44—C45—C46—C47	143.14 (16)
C10—C11—C29—C30	141.59 (17)	C44—C45—C46—C58	−0.1 (2)
C10—C26—C27—C28	−0.2 (2)	C45—C27—C28—C29	143.43 (16)
C10—C26—C27—C45	−138.81 (17)	C45—C27—C28—C47	0.55 (19)
C11—C10—C26—C25	−143.61 (16)	C45—C46—C47—C28	−0.40 (19)
C11—C10—C26—C27	−0.4 (2)	C45—C46—C47—C48	−143.13 (16)
C11—C12—C13—C14	142.50 (16)	C45—C46—C58—C57	0.2 (2)
C11—C12—C13—C30	0.24 (19)	C45—C46—C58—C59	138.44 (17)
C11—C29—C30—C13	−0.21 (19)	C46—C47—C48—C31	138.01 (17)
C11—C29—C30—C31	−142.90 (16)	C46—C47—C48—C49	0.0 (2)
C12—C2—C3—C4	145.45 (16)	C46—C58—C59—C49	0.6 (2)
C12—C2—C3—C15	0.0 (2)	C46—C58—C59—C60	−142.16 (16)
C12—C11—C29—C28	−142.02 (17)	C47—C28—C29—C11	138.52 (17)
C12—C11—C29—C30	0.36 (19)	C47—C28—C29—C30	0.5 (2)
C12—C13—C14—C15	0.4 (2)	C47—C46—C58—C57	−138.78 (17)
C12—C13—C14—C33	−138.16 (17)	C47—C46—C58—C59	−0.5 (2)
C12—C13—C30—C29	−0.02 (19)	C47—C48—C49—C50	142.08 (16)
C12—C13—C30—C31	142.73 (16)	C47—C48—C49—C59	0.0 (2)
C13—C14—C15—C3	−1.5 (2)	C48—C31—C32—C33	142.21 (16)
C13—C14—C15—C16	−142.98 (16)	C48—C31—C32—C50	0.15 (19)
C13—C14—C33—C32	0.2 (3)	C48—C49—C50—C32	0.40 (18)
C13—C14—C33—C34	142.75 (16)	C48—C49—C50—C51	−142.24 (16)
C13—C30—C31—C32	−0.1 (2)	C48—C49—C59—C58	−0.3 (2)
C13—C30—C31—C48	−138.13 (17)	C48—C49—C59—C60	137.81 (17)
C14—C13—C30—C29	−142.36 (16)	C49—C50—C51—C35	138.21 (17)
C14—C13—C30—C31	0.4 (3)	C49—C50—C51—C52	0.1 (2)
C14—C15—C16—C17	141.76 (16)	C49—C59—C60—C52	−0.3 (2)
C14—C15—C16—C34	−0.04 (18)	C49—C59—C60—C56	−143.10 (16)
C14—C33—C34—C16	0.27 (19)	C50—C32—C33—C14	137.67 (17)
C14—C33—C34—C35	−141.97 (16)	C50—C32—C33—C34	−0.5 (3)
C15—C3—C4—C5	157.35 (15)	C50—C49—C59—C58	−137.96 (17)
C15—C3—C4—C18	3.1 (2)	C50—C49—C59—C60	0.2 (2)
C15—C14—C33—C32	−142.82 (16)	C50—C51—C52—C53	143.05 (16)
C15—C14—C33—C34	−0.29 (19)	C50—C51—C52—C60	−0.2 (2)
C15—C16—C17—C18	1.7 (2)	C51—C35—C36—C37	141.93 (17)
C15—C16—C17—C37	−138.69 (17)	C51—C35—C36—C53	−0.30 (19)
C15—C16—C34—C33	−0.14 (19)	C51—C52—C53—C36	−0.70 (19)
C15—C16—C34—C35	142.33 (16)	C51—C52—C53—C54	−143.13 (17)
C16—C17—C18—C4	−1.2 (2)	C51—C52—C60—C56	138.83 (17)
C16—C17—C18—C19	−144.08 (16)	C51—C52—C60—C59	0.3 (2)
C16—C17—C37—C36	0.7 (2)	C52—C53—C54—C39	137.88 (17)
C16—C17—C37—C38	143.93 (16)	C52—C53—C54—C55	0.1 (3)
C16—C34—C35—C36	0.1 (2)	C53—C36—C37—C17	138.16 (17)
C16—C34—C35—C51	−138.11 (17)	C53—C36—C37—C38	−0.7 (3)
C17—C16—C34—C33	−141.61 (17)	C53—C52—C60—C56	0.1 (3)
C17—C16—C34—C35	0.9 (3)	C53—C52—C60—C59	−138.49 (17)
C17—C18—C19—C20	142.40 (16)	C53—C54—C55—C41	142.31 (16)
C17—C18—C19—C38	0.01 (18)	C53—C54—C55—C56	−0.1 (3)
C17—C37—C38—C19	0.74 (19)	C54—C39—C40—C21	141.86 (16)
C17—C37—C38—C39	−142.88 (16)	C54—C39—C40—C41	−0.04 (19)
C18—C4—C5—C1	134.54 (16)	C54—C55—C56—C57	138.24 (17)
C18—C4—C5—C6	12.8 (2)	C54—C55—C56—C60	0.0 (2)
C18—C4—C5—C61	−111.43 (17)	C55—C41—C42—C23	137.69 (17)
C18—C17—C37—C36	−143.93 (16)	C55—C41—C42—C43	−0.2 (2)
C18—C17—C37—C38	−0.74 (19)	C55—C56—C57—C43	0.1 (2)
C18—C19—C20—C6	13.7 (2)	C55—C56—C57—C58	−142.56 (16)
C18—C19—C20—C21	−136.52 (16)	C55—C56—C60—C52	0.0 (3)
C18—C19—C38—C37	−0.46 (18)	C55—C56—C60—C59	142.90 (16)
C18—C19—C38—C39	143.02 (16)	C56—C57—C58—C46	142.31 (16)
C19—C20—C21—C22	141.89 (16)	C56—C57—C58—C59	−0.26 (19)
C19—C20—C21—C40	−2.6 (2)	C57—C43—C44—C24	142.35 (16)
C19—C38—C39—C40	−1.3 (2)	C57—C43—C44—C45	−0.2 (2)
C19—C38—C39—C54	−138.89 (17)	C57—C56—C60—C52	−142.71 (16)
C20—C6—C7—C8	134.74 (16)	C57—C56—C60—C59	0.23 (19)
C20—C6—C7—C22	−17.85 (16)	C57—C58—C59—C49	143.16 (16)
C20—C6—C68—O1	153.44 (13)	C57—C58—C59—C60	0.40 (19)
C20—C6—C68—C67	−80.09 (16)	C58—C46—C47—C28	142.95 (16)
C20—C19—C38—C37	−142.50 (16)	C58—C46—C47—C48	0.2 (2)
C20—C19—C38—C39	1.0 (2)	C58—C59—C60—C52	142.44 (16)
C20—C21—C22—C7	0.40 (18)	C58—C59—C60—C56	−0.39 (19)
C20—C21—C22—C23	−145.40 (15)	C59—C49—C50—C32	142.55 (16)
C20—C21—C40—C39	2.2 (2)	C59—C49—C50—C51	−0.1 (2)
C20—C21—C40—C41	139.68 (17)	C60—C52—C53—C36	142.34 (17)
C21—C22—C23—C24	138.94 (17)	C60—C52—C53—C54	−0.1 (3)
C21—C22—C23—C42	1.2 (2)	C60—C56—C57—C43	142.69 (16)
C21—C40—C41—C42	1.1 (2)	C60—C56—C57—C58	0.02 (19)
C21—C40—C41—C55	−141.54 (16)	C61—O2—C74—C69	76.4 (2)
C22—C7—C8—C9	136.62 (16)	C61—O2—C74—C73	−104.46 (19)
C22—C7—C8—C25	−1.2 (2)	C61—C5—C6—C7	−125.72 (15)
C22—C21—C40—C39	−138.17 (16)	C61—C5—C6—C20	120.71 (15)
C22—C21—C40—C41	−0.7 (2)	C61—C5—C6—C68	−0.72 (19)
C22—C23—C24—C25	0.3 (2)	C61—C62—C63—C64	−178.36 (15)
C22—C23—C24—C44	−141.64 (16)	C61—C62—C67—C66	177.08 (15)
C22—C23—C42—C41	−0.8 (2)	C61—C62—C67—C68	−1.7 (2)
C22—C23—C42—C43	141.59 (16)	C62—C63—C64—C65	1.4 (3)
C23—C24—C25—C8	1.5 (2)	C62—C67—C68—O1	76.77 (18)
C23—C24—C25—C26	−138.04 (17)	C62—C67—C68—C6	−46.9 (2)
C23—C24—C44—C43	−0.49 (18)	C63—C62—C67—C66	−0.6 (2)
C23—C24—C44—C45	142.21 (16)	C63—C62—C67—C68	−179.43 (15)
C23—C42—C43—C44	−0.10 (18)	C63—C64—C65—C66	−0.6 (3)
C23—C42—C43—C57	−141.97 (16)	C64—C65—C66—C67	−0.8 (3)
C24—C23—C42—C41	−142.60 (16)	C65—C66—C67—C62	1.4 (2)
C24—C23—C42—C43	−0.21 (18)	C65—C66—C67—C68	−179.82 (15)
C24—C25—C26—C10	143.77 (16)	C66—C67—C68—O1	−102.01 (18)
C24—C25—C26—C27	0.3 (2)	C66—C67—C68—C6	134.35 (16)
C24—C44—C45—C27	−0.2 (2)	C67—C62—C63—C64	−0.8 (2)
C24—C44—C45—C46	−138.01 (17)	C68—O1—C69—C70	106.84 (17)
C25—C8—C9—C1	142.51 (16)	C68—O1—C69—C74	−74.07 (19)
C25—C8—C9—C10	0.04 (18)	C68—C6—C7—C8	−114.12 (17)
C25—C24—C44—C43	−142.09 (16)	C68—C6—C7—C22	93.29 (16)
C25—C24—C44—C45	0.6 (2)	C68—C6—C20—C19	112.37 (17)
C25—C26—C27—C28	138.73 (17)	C68—C6—C20—C21	−95.09 (15)
C25—C26—C27—C45	0.1 (2)	C69—O1—C68—C6	123.71 (15)
C26—C10—C11—C12	137.90 (17)	C69—O1—C68—C67	−1.1 (2)
C26—C10—C11—C29	0.9 (2)	C69—C70—C71—C72	−0.2 (3)
C26—C27—C28—C29	0.4 (3)	C70—C69—C74—O2	177.97 (15)
C26—C27—C28—C47	−142.49 (16)	C70—C69—C74—C73	−1.1 (3)
C26—C27—C45—C44	−0.2 (3)	C70—C71—C72—C73	−0.2 (3)
C26—C27—C45—C46	142.04 (16)	C71—C72—C73—C74	0.0 (3)
C27—C28—C29—C11	0.1 (3)	C72—C73—C74—O2	−178.38 (16)
C27—C28—C29—C30	−137.92 (17)	C72—C73—C74—C69	0.7 (3)
C27—C28—C47—C46	−0.09 (19)	C74—O2—C61—C5	−123.13 (15)
C27—C28—C47—C48	142.60 (16)	C74—O2—C61—C62	1.1 (2)
C27—C45—C46—C47	0.74 (19)	C74—C69—C70—C71	0.8 (2)
C27—C45—C46—C58	−142.53 (16)	S1^i^—S1—C75—S1^ii^	−132.40 (4)
C28—C27—C45—C44	−143.06 (16)	S1^i^—S1—C75—S1^iii^	132.40 (4)
C28—C27—C45—C46	−0.80 (19)		

Symmetry codes: (i) −*x*+1/2, −*y*+1/2, *z*; (ii) −*y*+1/2, *x*, −*z*+1/2; (iii) *y*, −*x*+1/2, −*z*+1/2.
